# Citron C-05 inhibits both the penetration and colonization of *Xanthomonas citri* subsp. *citri* to achieve resistance to citrus canker disease

**DOI:** 10.1038/s41438-020-0278-4

**Published:** 2020-05-01

**Authors:** Hongyan Fu, Mingming Zhao, Jing Xu, Limei Tan, Jian Han, Dazhi Li, Meijun Wang, Shunyuan Xiao, Xianfeng Ma, Ziniu Deng

**Affiliations:** 1 College of Horticulture, Hunan Agricultural University, 410128 Changsha, Hunan China; 2 National Center for Citrus Improvement, 410128 Changsha, Hunan China; 3 Hunan Horticultural Research Institute, 410125 Changsha, Hunan China; 4 Institute for Bioscience and Biotechnology Research & Department of Plant Sciences and Landscape Architecture, University of Maryland College Park, Rockville, MD 20850 USA

**Keywords:** Plant breeding, Biotic

## Abstract

Citrus canker, caused by *Xanthomonas citri* subsp. *citri* (*Xcc*), is a serious bacterial disease that affects citrus production worldwide. Citron C-05 (*Citrus medica*) is the only germplasm in the *Citrus* genus that has been identified to exhibit strong resistance to *Xcc*. However, it has not been determined when, where, and how *Xcc* is restricted in the tissues of Citron C-05 during the infection process. In the present study, we investigated the spatiotemporal growth dynamics of an *eGFP*-labeled virulent *Xcc* (*eGFP-Xcc*) strain in Citron C-05 along with five susceptible biotypes (i.e., lemon, pummelo, sour orange, sweet orange, and ponkan mandarin) upon inoculation via the spraying or leaf infiltration of a bacterial suspension. The results from extensive confocal laser scanning microscopy analyses showed that while *Xcc* grew rapidly in plants of all five susceptible genotypes, *Xcc* was severely restricted in the epidermal and mesophyll cell layers of the leaves of Citron C-05 in the early stage of infection. Not surprisingly, resistance against *Xcc* in Citron C-05 was found to be associated with the production of reactive oxygen species and hypersensitive response-like cell death, as well as greater upregulation of several defense-related genes, including a pathogenesis-related gene (*PR1*) and a glutathione S-transferase gene (*GST1*), compared with sweet orange as a susceptible control. Taken together, our results not only provide further valuable details of the spatiotemporal dynamics of the host entry, propagation, and spread of *Xcc* in both resistant and susceptible citrus plants but also suggest that resistance to *Xcc* in Citron C-05 may be attributed to the activation of multiple defense mechanisms.

## Introduction

Citrus canker is a major bacterial disease that affects most commercial citrus varieties worldwide^1^ and causes serious economic losses in almost all the major citrus-producing areas in China^2^. The causal agent is *Xanthomonas citri* subsp. *citri* (*Xcc*), a Gram-negative bacterial pathogen capable of colonizing all aboveground tissues of citrus plants, including young leaves, thorns, shoots, and fruits^3,4^. Under warm temperature and high-humidity conditions, the canker lesions first appear as circular oily spots, usually on the abaxial leaf surface, and then develop into tiny, slightly raised blister-like lesions^5^. The typical characteristic symptom is the formation of an enlarged hyperplastic lesion with a raised and spongy or corky center surrounded by a yellow chlorotic halo^4,6^, where the bacteria remain alive and active^3^ in the late stage of an infection. Severe canker disease causes defoliation, shoot dieback, and premature fruit drop^6,7^.

Different citrus species and cultivars exhibit different levels of sensitivity to canker disease. Susceptible biotypes of *Citrus* and its relatives include sweet orange (*Citrus sinensis*), pummelo (*C. grandis*), lemon (*C. limon*), lime (*C. aurantifolia*), grapefruit (*C. paradisi*), clementine (*C. clementina*), trifoliate orange (*Poncirus trifoliata*), and some mandarin-like hybrids such as “Orah” and “Orri”^3,7^. Certain citrus biotypes, such as mandarin (*C. reticulata*), Ichang papeda (*C. ichangenesis*), and *C. junos*, usually do not show canker symptoms in the field; however, when they are artificially inoculated with *Xcc*, they develop typical canker disease symptoms^8^. Kumquat (*Fortunella* spp.) and its hybrid calamondin (also known as *Citrofortunella*) are considered to be resistant to canker disease^9,10^. Through many years of efforts aimed at screening for *Xcc*-resistant citrus germplasm, we identified a citron (*C. medica*) biotype designated “Citron C-05” as highly resistant to *Xcc* in both field and laboratory conditions^8^. However, when, where and how *Xcc* bacteria are restricted in Citron C-05, the sole resistant citrus biotype reported thus far, have yet to be determined.

To establish successful colonization, *Xcc* bacteria have to penetrate host tissues through stomata or wounds. The length of the latent infection period is temperature dependent. It takes 6–7 days for the bacteria to propagate in several layers of mesophyll cells before the appearance of typical canker symptoms, including yellowing and crater formation, under warm temperature, high-humidity conditions^11,12^. Conceivably, resistance and tolerance in citrus hosts that do not display typical canker symptoms may involve distinct mechanisms. Preformed or inducible physical barriers may prevent bacterial attachment to the host surface and/or penetration into host tissues before colonization can occur^13^. Effector-triggered immunity (ETI), which is activated by resistance (R) proteins (normally members of the nucleotide-binding (NB) leucine-rich-repeat (LRR) superfamily), can restrict the propagation of bacteria within host tissue^14^. ETI is often, though not always, accompanied by a hypersensitive response (HR), consisting of pathogen-induced, localized programmed cell death at the site of infection^15^. In plants exhibiting tolerance, the bacterial pathogen can propagate in the host tissue, but the infection causes no or less-typical disease symptoms^16^. Additionally, the developmental stage of the plant or even the age of individual leaves may impact the degree of *Xcc* infection^17,18^; thus, the use of plants and leaves in similar developmental stages for the determination of *Xcc* infection in mesophyll tissues is considered to be critical for the reliable evaluation of germplasms for resistance or susceptibility to citrus canker^17,18^. For example, based on our observations, the leaves of a susceptible citrus plant may show reduced susceptibility to spray-inoculated *Xcc* when they turn dark green as they mature (data not shown), presumably because there are age-dependent structural changes such as wax deposition in the leaves that may restrict the epiphytic colonization of bacteria^4,18^. To more reliably and efficiently assess the resistance or susceptibility of host plants to a particular pathogen and infer likely associated mechanisms, fluorescence protein-labeled pathogen strains have been widely used for studying bacterial colonization and biofilm formation at a much improved spatiotemporal resolution^11,19,20^. We have thus also successfully developed an *eGFP*- *Xcc* strain with comparable pathogenicity to the wild-type strain DL509 isolated by our group^21^ for studying the *Xcc* infection process in citrus.

Based on our evaluations conducted in the past 10 years, Citron C-05 is the only resistant biotype in the genus *Citrus* that displays high resistance to canker disease in the field, as well as under greenhouse conditions upon natural infection or artificial inoculation with *Xcc*^8^. In this study, to determine the types of resistance mechanisms by which *Xcc* is restricted in Citron C-05, we conducted a comparative analysis of the *Xcc* infection process between Citron C-05 and five selected susceptible citrus biotypes by taking advantage of the availability of an *eGFP*-*Xcc* strain and a confocal imaging facility. Our results revealed the spatiotemporal dynamics of *Xcc* infection in both susceptible and resistant citrus biotypes and suggested that Citron C-05 can mount defense responses to effectively restrict *Xcc* propagation in mesophyll cells upon host penetration.

## Results

### *Xcc* growth and canker symptom development on the leaf surface

**Fig. 1:**
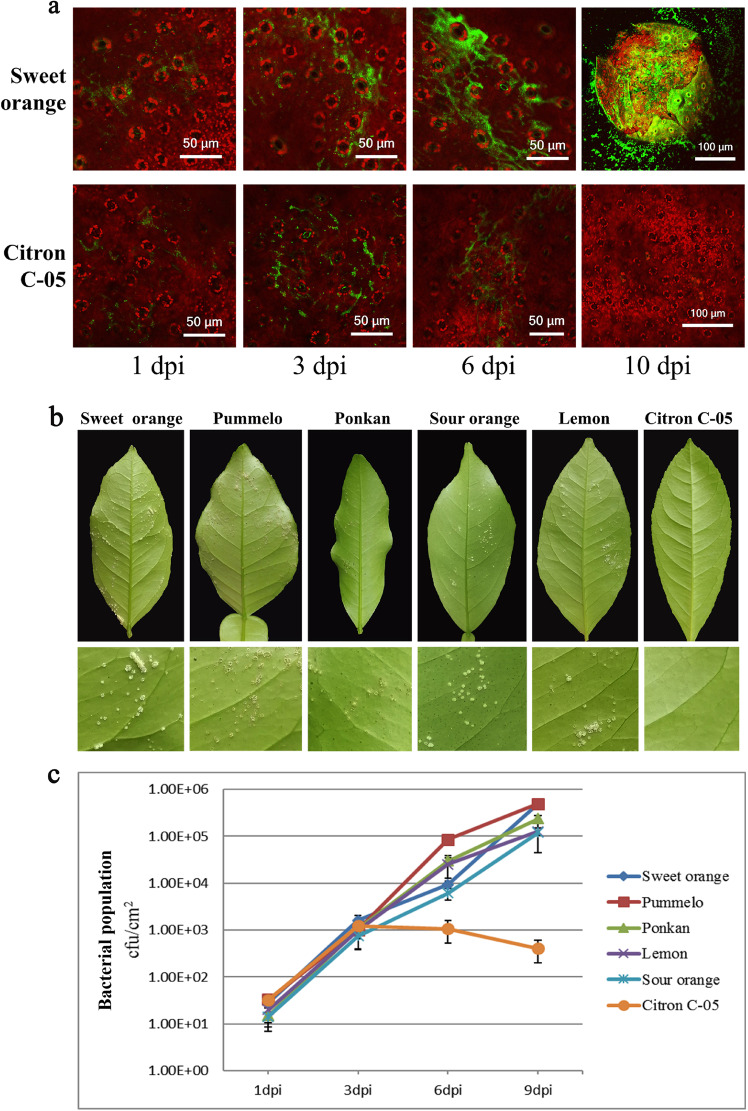
Leaf-surface bacterial growth and canker system development in citrus leaves spray-inoculated with *Xcc*. Leaves of plants from Citron C-05 and sweet orange (and four other biotypes, Supplementary Fig. S1) were sprayed with ~ 10^8^ cfu/ml *eGFP*-*Xcc* on the abaxial surface of fully expanded young leaves of six citrus biotypes. The inoculated leaves were subjected to confocal imaging for the observation of *Xcc* growth and the visual assessment of disease symptoms. These experiments were repeated at least three times with similar results. **a** Representative images showing the growth of *Xcc* on the leaf surface of sweet orange and Citron C-05 at the indicated time points. **b** Representative leaves (sections) showing the canker disease symptoms of the six indicated biotypes at 10 dpi. **c** Quantification of *Xcc* growth in the inoculated leaves of the six indicated citrus biotypes over a time course

It is believed that successful attachment, adaptation and colonization on the host surface are the first steps for bacterial pathogens such as *Xcc* to establish a successful infection in the host plant^22,23^. To assess whether Citron C-05 can inhibit *Xcc* surface attachment and leaf-surface growth, young leaves of a similar age from Citron C-05 and five citrus biotypes (i.e., sweet orange, lemon, ponkan, pummelo, and sour orange) known to be susceptible to *Xcc* were inoculated by spraying a 10^8^ cfu/ml *eGFP*-*Xcc* suspension on the abaxial leaf surface. Bacterial growth was monitored with a Zeiss confocal laser scanning microscope 710 (CLSM 710) at multiple time points after inoculation. From 1 to 3 days post-inoculation (dpi), similar bacterial growth on the leaf surface, including the intercellular space of the epidermal cells, was observed in all six citrus biotypes (Fig. 1a and Supplementary Fig. S1), suggesting that bacterial attachment and growth on the epidermal surface are not noticeably inhibited in Citron C-05. At 6 dpi, however, while *Xcc* continued to grow on the five susceptible biotypes, as shown by a significant increase in eGFP fluorescence (Fig. 1a and Supplementary Fig. S1), no further bacterial growth was observed on Citron C-05, whose leaves often showed even less eGFP fluorescence compared to the same leaves at 3 dpi (Fig. 1a). Z-stack images were generated to assess the distribution of bacteria in the spray-inoculated leaves of sweet orange and Citron C-05 at 6 dpi. As shown in Supplementary Fig. S4, *Xcc* bacteria were mostly detected in the intercellular space between the epidermal cells and some substomatal chambers on the leaf surface of sweet orange; however, *Xcc* bacteria were rarely or only sporadically observed on the leaf surface of Citron C-05. At 10 dpi, typical canker symptoms and intense fluorescence signals from *eGFP*-*Xcc* were detected in all five susceptible biotypes (Fig. 1a, b and Supplementary Fig. S1). By contrast, no canker symptoms were visible in Citron C-05 (Fig. 1b). Strikingly, eGFP fluorescence was hardly detectable either on the leaf surface or in mesophyll layers of Citron C-05 (Fig. 1a). Altogether, these observations suggest that resistance against *Xcc* in Citron C-05 leaves mostly occurs inside the mesophyll tissues at a post-penetration stage after 3 dpi.

### Quantification and vertical distribution of *Xcc* after leaf tissue penetration

After successful penetration into the leaf tissue, especially the mesophyll layer, *Xcc* bacteria have to suppress host defense to colonize and proliferate in the apoplast of the host tissue, eventually leading to canker symptom development. To confirm that resistance to *Xcc* in Citron C-05 indeed occurs inside the infected tissue, we quantified *Xcc* growth in inoculated leaves after surface sterilization with 75% ethanol. No significant differences in bacterial growth were detected among the six citrus biotypes at 1 or 3 dpi (Fig. 1c). However, while *Xcc* continued to proliferate in all five susceptible citrus biotypes, reaching ~ 10^5^ cfu/ml, *Xcc* stopped proliferating inside Citron C-05 leaf tissue at 3 dpi, and the bacterial titer actually decreased to 4 × 10^2^ cfu/ml at 9 dpi, corresponding to ~ 1000× fewer bacteria compared to the bacterial levels in the other biotypes (Fig. 1c). These results agreed well with both the amount of *Xcc* visualized by confocal imaging and canker symptom development on the leaf surface (Fig. 1a, b).

**Fig. 2:**
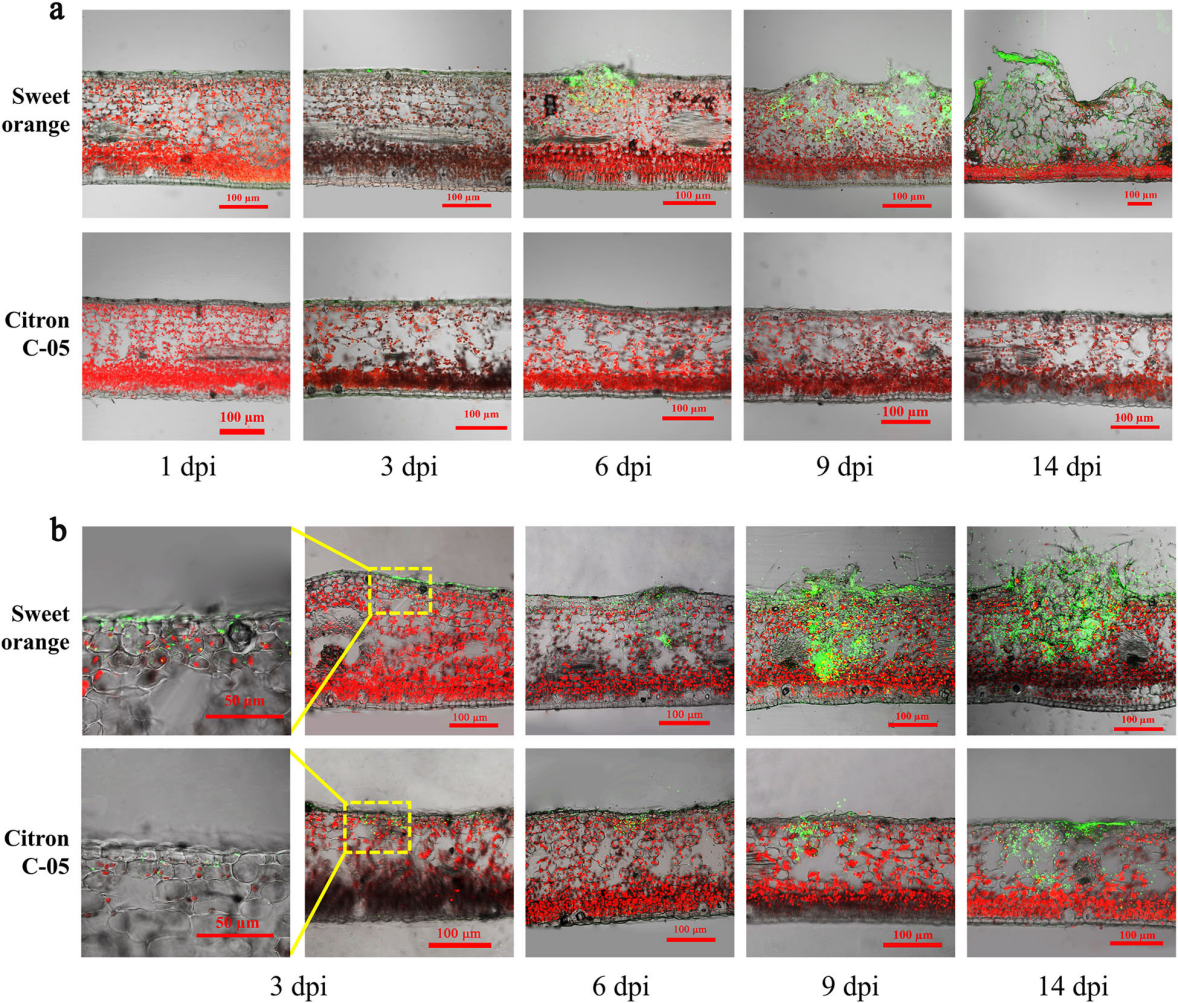
Spatiotemporal growth dynamics of *Xcc* visualized by CLSM in vertical sections of spray-inoculated leaves of sweet orange and Citron C-05. **a** Fully expanded young leaves of the indicated biotypes were inoculated by spraying 10^8^ cfu/ml *eGFP*-*Xcc*. **b** Younger leaves (half the size of fully expanded leaves) were sprayed with 10^9^ cfu/ml *eGFP*-*Xcc*. Leaf vertical sections were monitored using CLSM. Representative images are shown

To further validate the above results, we examined the spatiotemporal distribution of *Xcc* in vertical sections of the inoculated leaves of Citron C-05 and “Bingtang” sweet orange, as a representative susceptible biotype, by CLSM. At 3 dpi with a bacterial suspension of 10^8 ^cfu/ml sprayed on fully expanded young leaves, some weak green fluorescence of *Xcc* was visible in mesophyll cells immediately underneath the epidermis in both “Bingtang” (more) sweet orange and Citron C-05 (less) (Fig. 2a). However, at 6 dpi, while large numbers of *Xcc* (reflected by more intense green fluorescence) were clearly visible in sweet orange leaf sections, no *Xcc* (reflected by the lack of fluorescence) was detectable in Citron C-05. Typical symptomatic crater structures concomitant with the spread and release of *Xcc* were visible in the leaf sections of the sweet orange at 9 dpi, which became more obvious at 14 dpi, whereas no leaf structural changes were seen in Citron C-05 (Fig. 2a). In an enhanced assay in which younger leaves (only half the size of fully expanded leaves) were sprayed with 10^9^ cfu/ml *Xcc*, more fluorescent *Xcc* were visible in the leaf sections of Citron C-05 at 3 dpi, which continued to increase over time, reaching a considerable level (as reflected by the increased amount of green fluorescence) by 14 dpi, yet there was still no crater formation observed (Fig. 2b). By contrast, at 9 and 14 dpi, there was a massive explosion of *Xcc* with the rupture of the craters in the leaf sections of sweet orange (Fig. 2b). These observations support the conclusion that Citron C-05 exhibits post-penetration resistance against *Xcc* and imply that *Xcc* fails to manipulate host cell growth and development in Citron C-05 to form craters as it does in susceptible citrus biotypes, even though it accumulates at a fairly high level upon artificial inoculation at a high concentration.

### *Xcc* infection phenotypes of six citrus biotypes recapitulated by infiltration inoculation

**Fig. 3:**
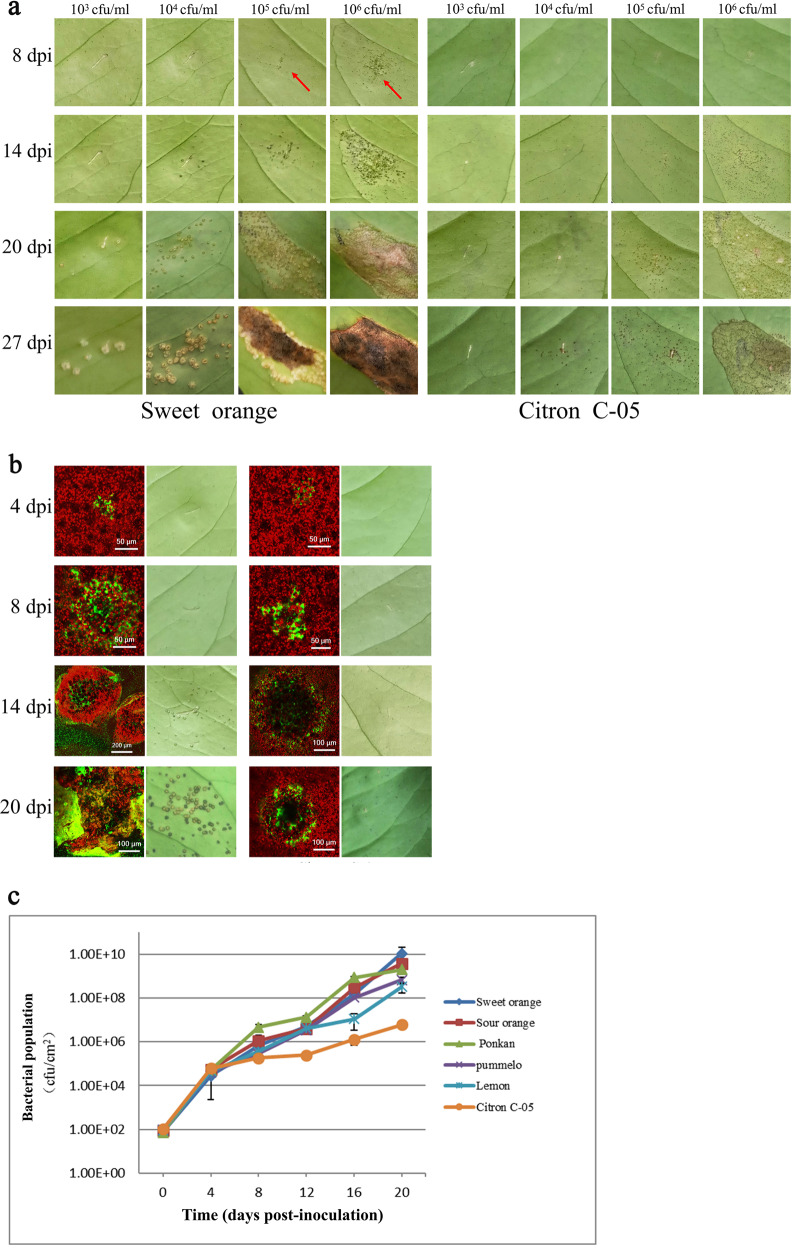
Infection phenotypes of sweet orange and Citron C-05 following infiltration inoculation. *Xcc* bacterial suspensions at different concentrations (10^3^, 10^4^, 10^5^, and 10^6^ cfu/ml) were infiltrated from the abaxial surface into fully expanded young leaves of the six citrus biotypes, and disease symptoms were monitored and documented from 1 to 27 dpi. Images of sweet orange and Citron C-05 are shown here. Images of the other biotypes are shown in Supplementary Fig. S2. **a** *Xcc* infection symptoms of sweet orange and Citron C-05 leaves infiltrated with the four indicated *Xcc* concentrations at the four indicated time points. **b** Visualization of *Xcc* after infiltration (at 10^4^ cfu/ml) of the leaves of sweet orange and Citron C-05 by CLSM at the indicated time points. The infection symptoms of the same leaves were photographed and are shown in the right panels. **c** Quantification of *Xcc* in the leaves of the six citrus biotypes infiltrated with 10^4^ cfu/ml *Xcc*

To evaluate the relationship between the bacterial load (i.e., quantity) and canker symptom development and to further assess the resistance level of Citron C-05 relative to those of the susceptible biotypes, we infiltrated the leaves of the six biotypes with *Xcc* bacterial suspensions at different concentrations (10^3^, 10^4^, 10^5^, and 10^6^ cfu/ml). Small blister-like lumps on the leaves appeared as the earliest symptom visible to the naked eye at ~8 dpi in all five susceptible genotypes, including sweet orange, when infiltrated with 10^6^ cfu/ml *Xcc*. These lumps gradually spread and merged to produce water-soaked symptoms at 20 dpi and became erumpent and necrotic, with yellow margins surrounding the infected sites, at 27 dpi (Fig. 3a and Supplementary Fig. S2). Small blister-like lumps also occurred on the leaves of sweet orange, ponkan and sour orange infiltrated with 10^5^ cfu/ml *Xcc* at 8 dpi, but at a lower density compared to the leaves infiltrated with 10^6 ^cfu/ml *Xcc* (Fig. 3a and Supplementary Fig. S2). Small cankers were visible on these three biotypes at 14 dpi and coalesced to form larger necrotic lesions with yellow margins at 20 dpi, which became more severe at 27 dpi. By contrast, the leaves of Citron C-05 showed only tiny black spots at 14 dpi, 20 dpi, and even 27 dpi, and there was no canker formation or significant necrosis when Citron C-05 leaves were infiltrated with *Xcc* at these two bacterial concentrations (Fig. 3a).

When the leaves of the six biotypes were infiltrated with 10^3^ and 10^4^ cfu/ml *Xcc*, small blister-like lumps (at a lower density) appeared 4–6 days later on the susceptible biotypes, and typical canker lesions were visible at 27 dpi (Fig. 3a). By contrast, no symptoms were visible in the leaves of Citron C-05 infiltrated with 10^3^ or 10^4^ cfu/ml *Xcc* except for small black spots at 27 dpi in the case of infiltration with 10^4^ cfu/ml *Xcc* (Fig. 3a). These results were further validated by CLSM of leaf sections of Citron C-05 and sweet orange (Fig. 3b) along with four other susceptible biotypes (Supplementary Fig. S2) infiltrated with 10^4^ cfu/ml *Xcc*.

To further evaluate the relationship between the growth dynamics of *Xcc* in citrus leaf tissues and canker symptom development, we quantified *Xcc* in the leaves of the six biotypes upon infiltration with 10^4^ cfu/ml *Xcc*. At 4 dpi, the bacteria grew almost equally well in the tissues of all the tested citrus biotypes, reaching a concentration of 10^4^ cfu/cm^2^. At 8 dpi, *Xcc* reached a concentration of 1.8 × 10^5^ cfu/cm^2^ in Citron C-05, whereas the five susceptible biotypes showed higher concentrations, ranging from 3.0 × 10^5^ to 4.7 × 10^6 ^cfu/cm^2^. The differences in bacterial growth between Citron C-05 and the other five biotypes continued to increase at 12 and 16 dpi, reaching a maximum of ~ 1000× at 20 dpi, with Citron C-05 exhibiting a bacterial load of 5.9 × 10^6^ cfu/cm^2^, and the loads in the five susceptible biotypes reaching 10^8^–10^9^ cfu/cm^2^ (Fig. 3c). Additionally, the bacterial load seemed to be positively correlated with canker disease severity among the susceptible biotypes; differences were first observed among the susceptible biotypes at 8 dpi and became more obvious at 20 dpi, with sweet orange, sour orange and ponkan being more susceptible than pummelo and lemon (Fig. 3c).

**Fig. 4:**
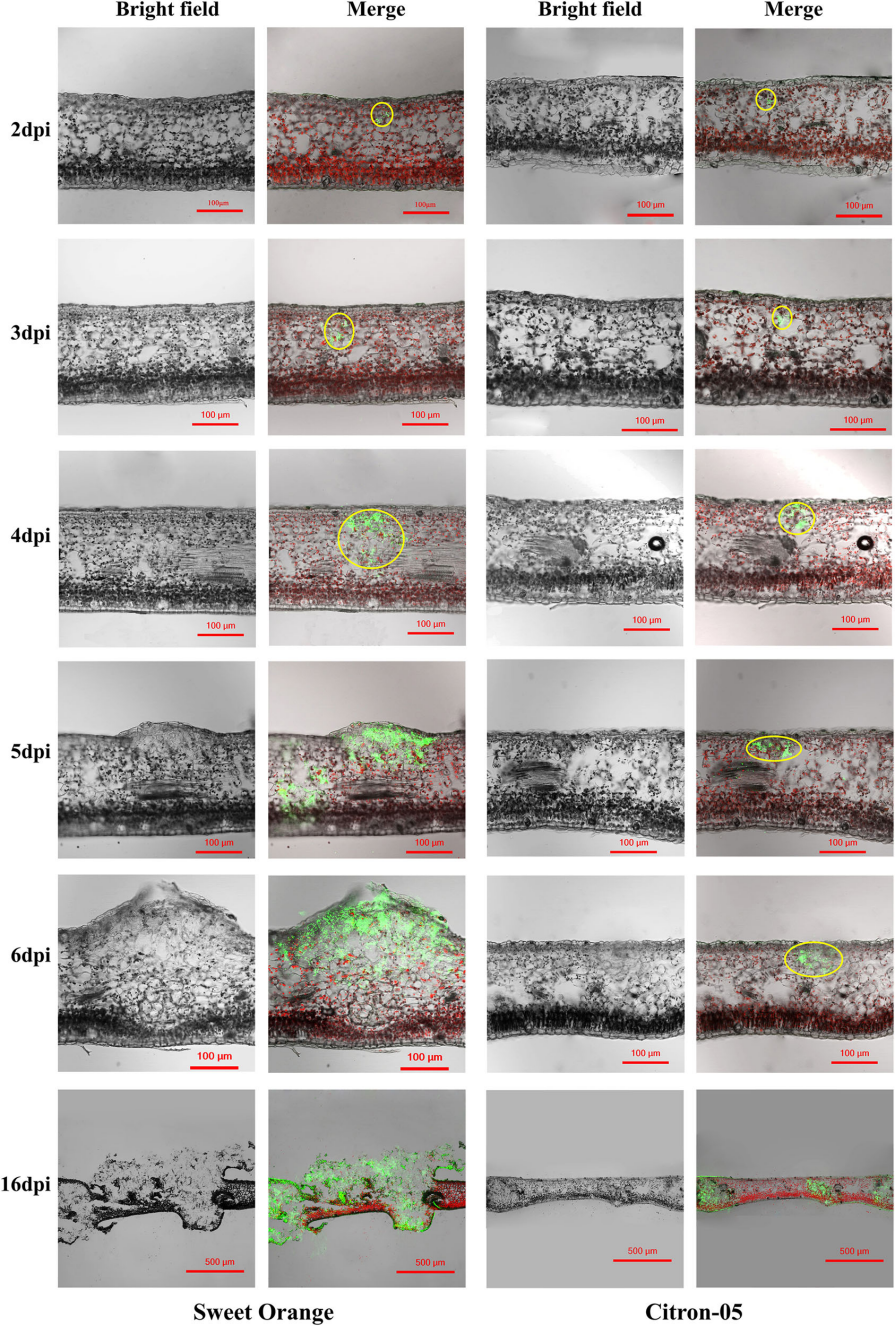
CLSM of leaf vertical sections showing *Xcc* proliferation in the leaves of sweet orange and Citron C-05 infiltrated with 10^5^ cfu/ml *eGFP-Xcc*. The yellow circles indicate the areas where *Xcc* aggregated

CLSM of vertical sections of the leaves of Citron C-05 and “Bingtang” sweet orange inoculated with 10^5^ cfu/ml *eGFP*-*Xcc* showed that there was no clear difference between these two biotypes at 2 dpi (Fig. 4). However, starting from 3 dpi, more *Xcc* was observed in “Bingtang” sweet orange than in Citron C-05, and the difference became more dramatic as time went on. In the leaves of “Bingtang” sweet orange, *Xcc* penetrated deep into the spongy tissue at 4 dpi, spread across the entire leaf vertical section at 5 dpi, and formed a crater at 6 dpi; the craters ruptured, destroying the local infected tissues at 16 dpi (Fig. 4). By contrast, in the leaves of Citron C-05, *Xcc* exhibited limited proliferation, and there was no crater formation even at 16 dpi, when *Xcc* had spread across the leaf vertical sections (Fig. 4). These results are in accord with our observations following spray inoculation and further support the hypothesis that Citron C-05 restricts *Xcc* proliferation after penetration into leaf mesophyll layers.

### Resistance to *Xcc* infection in Citron C-05 is associated with the induction of defense-related genes

**Fig. 5:**
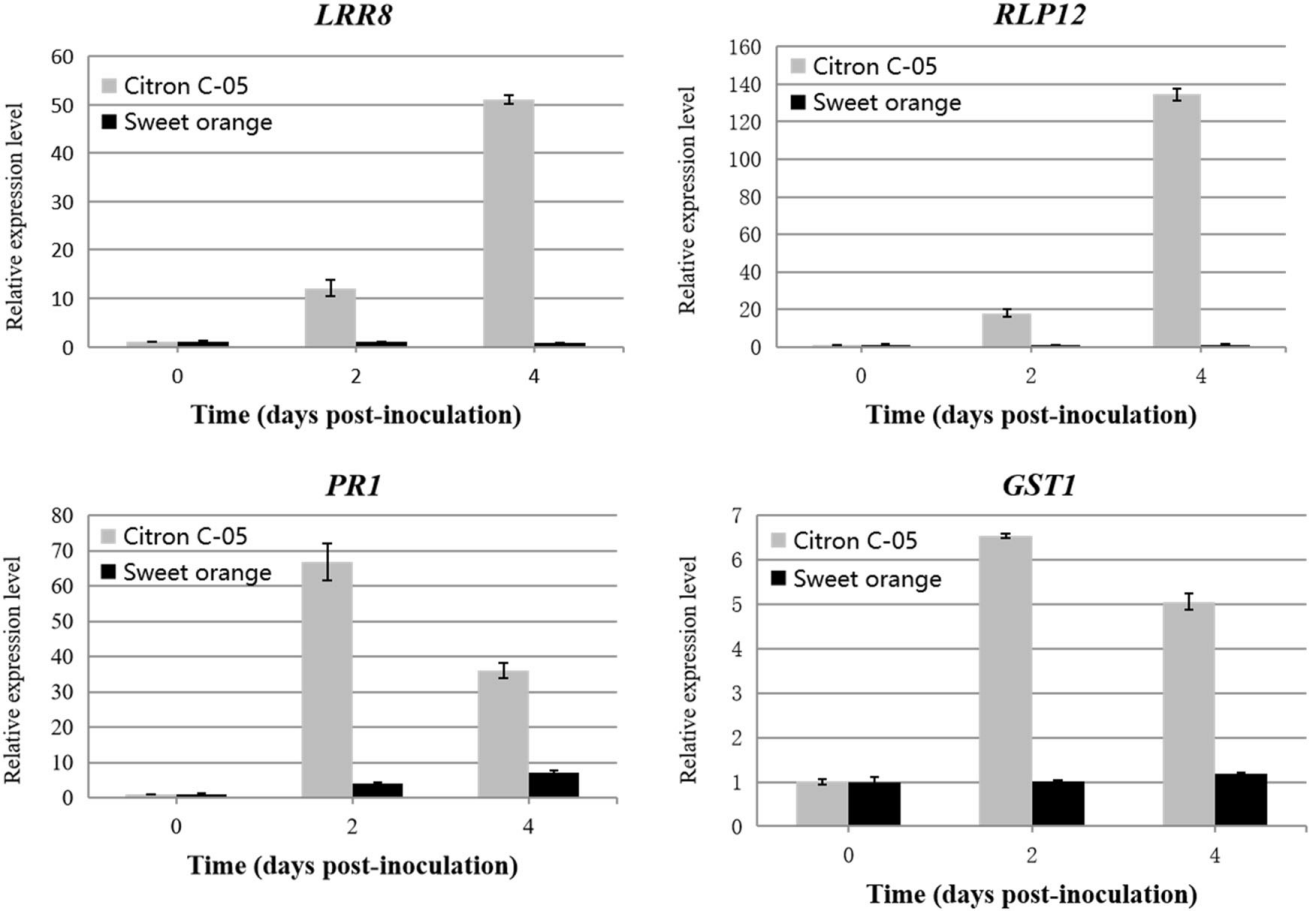
Induction of four defense-related genes in Citron C-05 upon *Xcc* inoculation. Quantitative RT-PCR was used to check the expression levels of the four indicated genes in the leaves of Citron C-05 and sweet orange infiltrated with a 10^5^ cfu/ml *Xcc* suspension at 0, 2, and 4 dpi

**Fig. 6:**
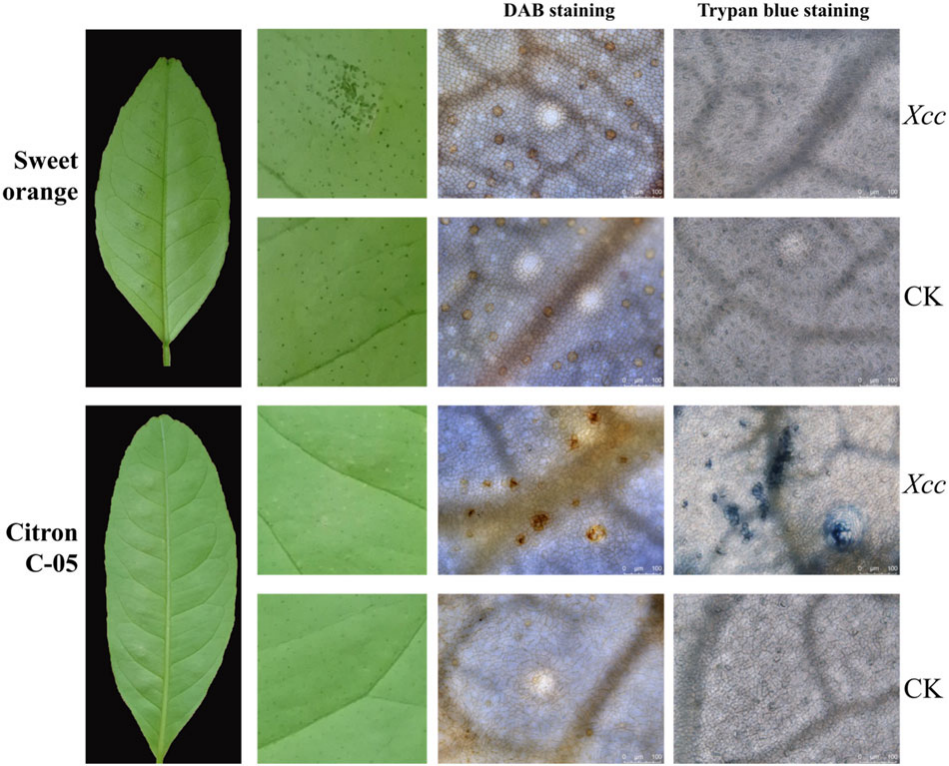
*Xcc* induces ROS accumulation and cell death in Citron C-05 cells. The leaves of Citron C-05 and sweet orange were infiltrated with 10^5^ cfu/ml *Xcc* or buffer. At 5 dpi, infiltrated leaves were subjected to DAB and Trypan blue staining. Leaf sections were examined under a Leica DMi8 scope. Reddish-brown staining indicates ROS accumulation, and blue staining indicates dead or dying cells

To assess the possible defense mechanisms activated in Citron C-05 when attacked by *Xcc*, we checked the expression of a number of genes whose homologs in model plants are known to be involved in disease resistance^24,25^. We infiltrated the leaves of Citron C-05 and Sweet orange with 10^5^ cfu/ml *Xcc* and measured the expression of *PR1* (encoding a pathogenesis-related protein), *RLP12* (encoding an LRR receptor-like protein), and *LRR8* (encoding an LRR receptor-like protein kinase) by quantitative reverse transcription PCR (RT-PCR). Consistent with the disease infection phenotypes, *PR1* was strongly induced (~67×) in Citron C-05 at 2 dpi, while it showed only a slight induction (~4×) in sweet orange (Fig. 5). Although the expression level of *PR1* decreased by approximately half in Citron C-05 at 4 dpi, it was still significantly higher (~4×) than that in sweet orange. More interestingly, while *RLP12* and *LRR8* were highly induced in Citron C-05, they were not induced in sweet orange at all (Fig. 5). Similarly, *GST1*, which is known to be involved in reactive oxygen species (ROS)-dependent defense^26^, was significantly upregulated in *Xcc*-infected Citron C-05 leaves but not in sweet orange (Fig. 5), suggesting that there may be massive ROS production in the *Xcc*-infected leaf tissues of Citron C-05. We thus examined ROS production in situ by DAB staining and found sporadic and localized ROS accumulation in the *Xcc-*inoculated leaves of Citron C-05 but not in those of sweet orange at 5 dpi (Fig. 6). ROS production often precedes programmed cell death in resistance gene-mediated resistance^27,28^. To determine whether the induction of ROS by *Xcc* in the leaves of Citron C-05 indeed leads to HR, we stained the leaves with trypan blue. As expected, small dead or dying cell clusters stained blue were also visible at 5 dpi only in *Xcc*-infected Citron C-05 leaves but not in infected sweet orange leaves (Fig. 6). These observations suggest that the resistance of Citron C-05 to *Xcc* may be attributable to the activation of multiple defense mechanisms.

## Discussion

In this study, we conducted a detailed spatiotemporal comparative analysis of the *Xcc* infection dynamics in five susceptible and one resistant citrus biotype. Our results showed that Citron C-05, the only resistant germplasm identified to date in the Citrus genus^8^, exhibited post-penetration resistance that was associated with the induction of at least four defense-related genes, ROS production and programmed cell death in most cases.

Type A *Xcc* is the main type causing severe symptoms in citrus orchards in Asian regions^29,30^. By using *eGFP*-*Xcc* pathotype A^21^ and CLSM as well as conventional means of bacterial growth determination and symptom examination, we were able to monitor the leaf-surface multiplication, penetration and proliferation inside the host tissue of *Xcc* in the leaves of six selected citrus biotypes. It has been well established that the stomata, as natural openings on the leaf surface, are exploited by pathogens to enter into host tissue and are thus subjected to tight regulation as part of a plant innate immune mechanism for restricting the entry of bacteria^31^. The results obtained via spray or infiltration inoculation showed that the inoculated leaves of all five susceptible biotypes exhibited typical canker symptoms (i.e., crater formation), whereas no crater formation was found in the inoculated leaves of Citron C-05, confirming that Citron C-05 is highly resistant to canker disease^8^. However, we noticed some differences in the kinetics of *Xcc* growth in Citron C-05 leaves inoculated with these two methods. *Xcc* growth in Citron C-05 leaves inculcated by spraying 10^8^ cfu/ml *Xcc* was completely arrested after 3 dpi, and a decreasing amount of the bacteria was over time (Fig. 1c), while *Xcc* that infiltrated into the leaf tissue multiplied ~100× in the period from 4 dpi to 20 dpi (Fig. 3c). In addition, when a high concentration (10^5^ cfu/ml) of *Xcc* was infiltrated into the leaves of Citron C-05, the bacteria proliferated to a greater extent at 16 dpi, as shown by CLSM images in Fig. 4. Altogether, these results suggest that while Citron C-05 is able to restrict *Xcc* inside leaf tissue to prevent its massive proliferation, the overall resistance of Citron C-05 to *Xcc* may in part be attributable to its ability to inhibit the leaf-surface multiplication and stomatal penetration of *Xcc*.

Mandarin is usually considered to be moderately resistant to *Xcc*^6^, and ponkan, as a hybrid of mandarin × pomelo, exhibits a low level of susceptibility to *Xcc*^17,32,33^. We found that ponkan was very susceptible in the experiments using either spray or infiltration inoculation in this study. This discrepancy might be caused by the absolute initial amount of *Xcc* that entered the inoculated leaf tissue, regardless of the specific inoculation method, even though leaf infiltration has been shown to induce canker disease development more readily^5^. In spray inoculation, because 10^4^ cfu/ml is reported to be the minimum inoculum concentration for causing symptom development in intact leaves that are less than 75% expanded^34^, a much higher *Xcc* concentration is often used to ensure more even disease development^33,35^. We thus used *Xcc* suspensions with concentrations as high as 10^8^ cfu/ml for spray inoculation. At such a high dose of inoculum, one may speculate that many more *Xcc* bacteria penetrate the leaf mesophyll layers of ponkan, causing typical canker symptoms. Since the infiltration of 10^3 ^cfu/ml or higher doses of *Xcc* also induced typical symptoms in all five susceptible citrus biotypes, we can further reason that (i) mandarin and some of its hybrids, such as ponkan, may possess relatively higher levels of stomatal immunity, capable of limiting *Xcc* entry into the leaf mesophyll under natural growth conditions when the amount of *Xcc* inoculum is rather low and heterogeneous or when leaves are inoculated with lower concentrations of *Xcc* and that (ii) ponkan, unlike Citron C-05, does not exhibit post-penetration resistance. Therefore, caution needs to be exercised when we evaluate the resistance and susceptibility of different citrus germplasms treated with different dosages of *Xcc*. Nevertheless, we considered spraying 10^8^ cfu/ml or infiltrating 10^3^ cfu/ml *Xcc* to be a safer inoculation method for revealing the distinct nature of the resistance of Citron C-05, which will be particularly useful for determining the phenotypes of segregating progenies derived from Citron C-05 x sweet orange for mapping resistance gene(s) in Citron C-05.

Kumquat and calamondin are considered to be resistant to canker disease, and the defense response to *Xcc* is associated with programmed cell death, H_2_O_2_ production and the induction of defense-related genes^7,9,10^. Currently, the genetic control mechanism of resistance to *Xcc* in Citron C-05 (as well as Kumquat and calamondin) is not known, nor is its molecular basis. The observed strong induction of four different defense-related genes (Fig. 5) is compatible with a scenario in which multiple layers of resistance may exist in Citron C-05. Specifically, while the induction of *LRR8* and *RLP12*, two genes encoding proteins involved in the perception of pathogen-associated molecular patterns (PAMPs)^24^, is suggestive of the activation of pattern-triggered immunity (PTI) in Citron C-05 (Fig. 5), the strong induction of *PR1* may imply the existence of *R* gene-mediated, salicylic acid-dependent resistance and systemic acquired resistance^25,36^. Consistent with the latter speculation, the resistance of Citron C-05 was also associated with *GST1* induction, ROS production and HR-like programmed cell death (Figs. 5 and 6). Future efforts will be directed toward the mapping and identification of the gene(s) underlying the remarkable resistance of Citron C-05 against *Xcc*.

## Materials and methods

### Plant materials

The genotypes used in the present study were “Citron C-05” (*C. medica*), “Zaomi” ponkan (*C. reticulate*), “Bingtang” sweet orange (*C. sinensis*), sour orange (*C. aurantium*), lemon (*C. limon*) and “Shatian” pummelo (*C. grandis*). All the citrus plants were grafted onto trifoliate orange (*Poncirus trifoliata*) rootstock and maintained for 2 years in the greenhouse of the National Center for Citrus Improvement, Changsha, China under the same conditions with standard irrigation and fertilization. Almost fully expanded young leaves from healthy plants were chosen for pathogenicity assays.

### *Xcc* culture and pathogenicity assays

The original *Xcc* strain DL509 used in this study was isolated from diseased sweet orange leaves showing typical cankers. *eGFP*-*Xcc* was then generated by triparental mating, and the bacterial expression of eGFP was confirmed by fluorescence microscopy and pathogenicity tests, indicating that the *eGFP*-*Xcc* strain exhibited the same pathogenicity as the wild-type strain^21^. *eGFP*-*Xcc* was plated on Luria-Bertani (LB) solid medium and incubated at 28 °C for 14 h. Individual colonies were cultured separately in LB liquid medium at 28 °C on a shaker at 200 rpm for 18 h. Bacterial cells were harvested and centrifuged at 8000 rpm at room temperature. The cell pellets were resuspended in sterile distilled water, and the concentration was measured by using a spectrophotometer at 600 nm. The concentration of the bacterial suspension was further confirmed by counting colonies on LB plates following appropriate dilution. The inocula were adjusted to concentrations of 10^3^, 10^4^, 10^5^, and 10^6^ cfu/ml for infiltration and 10^8^ cfu/ml for spray inoculation.

Spray inoculation was performed by uniformly distributing a 10^8^ cfu/ml bacterial suspension with 0.05% Silwet-L7 on the abaxial leaf blade. Infiltration inoculation was also performed on the abaxial surface. The leaves were carefully wounded with a needle, and then four concentrations of *Xcc* suspensions were separately infiltrated into the mesophyll of the same leaf by pressure using a 1 ml syringe. Distilled water was used as a control in both spray and infiltration inoculation. The inoculated plants were kept in a greenhouse under a controlled environment at ≥28 °C with ≥70% relative humidity. Lesion expansion and symptom development were recorded periodically after inoculation.

### Periodic quantification of *Xcc* in the host mesophyll after inoculation

Leaves inoculated by spray inoculations (10^8^ cfu/ml) and infiltration inoculation (10^4^ cfu/ml) were randomly sampled for quantification. Spray-inoculated leaves were collected at 1, 3, 6, and 9 days post-inoculation (dpi), and infiltration-inoculated leaves were collected at 0, 4, 8, 12, 16, and 20 dpi. The sampled leaves were surface disinfected with 75% ethanol. Eight leaf discs were collected within the inoculation area from each of the three inoculated leaves and homogenized in 1 ml of sterile distilled water. The isolation suspension was gradient diluted with sterile distilled water. Then, 20 μl of a diluted suspension from each sample was incubated on plates with solid LB medium at 28 °C for 48 h. The bacteria were quantified according to the formation of colonies on the LB agar plates. All quantifications were repeated three times.

### Monitoring the penetration and colonization of *Xcc* in citrus tissues

The penetration and colonization of *Xcc* in vivo was examined following the inoculation of *eGFP*-*Xcc* and observed under an inverted confocal laser scanning microscope as described previously^21^. Spray inoculation was performed at a concentration 10^8^ cfu/ml on fully expanded young leaves or 10^9^ cfu/ml on 1/2 expanded young leaves, and 10^5^ cfu/ml *Xcc* was directly infiltrated into fully expanded young leaves. Six rectangular leaf pieces 0.5 × 1.0 cm^2^ were sampled from each inoculated leaf and directly frozen in a cryostat (Leica CM1900, Berlin, Germany) at −20 °C. In addition, 10 μm serial frozen sections were cut along the vertical discs and blocked in 20% glycerol (v/v). For bacterial observations, eGFP fluorescence was imaged at an excitation wavelength of 488 nm and emission wavelength of 500–530 nm. The autofluorescence signal from chlorophyll was collected simultaneously at light wavelengths between 650 and 700 nm.

### *Xcc* growth and disease development on the leaf surface and in the mesophyll

*eGFP*-*Xcc* was inoculated onto fully expanded young leaves by spraying (10^8^ cfu/ml) and infiltration (10^4 ^cfu/ml) to monitor bacterial growth and disease development. The inoculated plants were maintained in a greenhouse at 27 ± 1 °C with high humidity. The presence of bacteria was examined under a Zeiss 710 confocal laser scanning microscope (CLSM) at ×10 magnification, and canker disease symptoms were recorded at regular intervals. Disease development after spray inoculation was observed at 1, 3, 6, and 10 dpi, and the bacterial colonization of *Xcc* was monitored at 4, 8, 14, and 20 dpi. Four leaves were sampled from each citrus genotype, and more than three discs of ~0.5 cm^2^ were cut from the sampled leaves. Then, the abaxial leaf surface was mounted for viewing under glass coverslip and observed with a Zeiss 710 confocal laser scanning microscope.

### ROS and trypan blue staining

Fully expanded young healthy leaves were infiltrated with 10^5^ cfu/ml *Xcc* bacteria. DAB (3,3’-diaminobenzidine) staining was performed for ROS analysis as reported by Piazza et al.^37^. The infiltration-inoculated leaves were submerged in a 1% (w/v) DAB solution and shaken at 100 rpm overnight. The leaves were cleaned in ethanol and excised at 5 dpi for DAB staining and destaining. Then, the leaf samples were examined for brown coloration under a Leica DMi8 microscope (Germany). Programmed cell death was visualized in inoculated leaves after staining with trypan blue at 5 dpi as described by Xiao et al.^27^.

### RNA extraction and real-time quantitative PCR analysis

The expression of defense genes (*PR1*, *RLP12*, *GST1*, and *LRR8*) induced by *Xcc* was evaluated. Young leaves were infiltrated with 10^5^ cfu/ml *Xcc*, and leaves were collected at 0, 2 and 4 dpi. Total leaf RNA was isolated using TRIzol reagent (Invitrogen, Grand Island, NY, USA) according to the protocol described previously^38^, and the RNA concentration was determined by using a Nanodrop 1000 spectrophotometer (Thermo Scientific, Wilmington, DE, USA). The synthesis of complementary DNA (cDNA) was performed with oligo (dT) primers using the M-MLV reverse transcriptase system (Promega, USA) according to the manufacturer’s instructions. The cDNA products were used for quantitative real-time PCR analysis in a Bio-Rad CFX96 qPCR system. All qPCR assays were performed in duplicate using the SYBR Green protocol. Primer sequences for *PR1*, *RLP12*, *GST1*, and *LRR8* were selected from the reported literature^24,25^.

### Data analysis

All of the presented data are shown as average values and standard errors. Differences between the means were evaluated using one-way analysis of variance.

## Supplementary information

Supplemental Information
