# Supplementary material for: Citron C-05 inhibits both the penetration and colonization of Xanthomonas citri subsp. citri to achieve resistance to citrus canker disease
Source: Hortic Res. 2020 May 1;7:58. doi: 10.1038/s41438-020-0278-4 (PMC7193574; doi:10.1038/s41438-020-0278-4)
Supplement: Supplementary file 1 — Supplemental Information [file 41438_2020_278_MOESM1_ESM.docx]

**Supplemental Information**


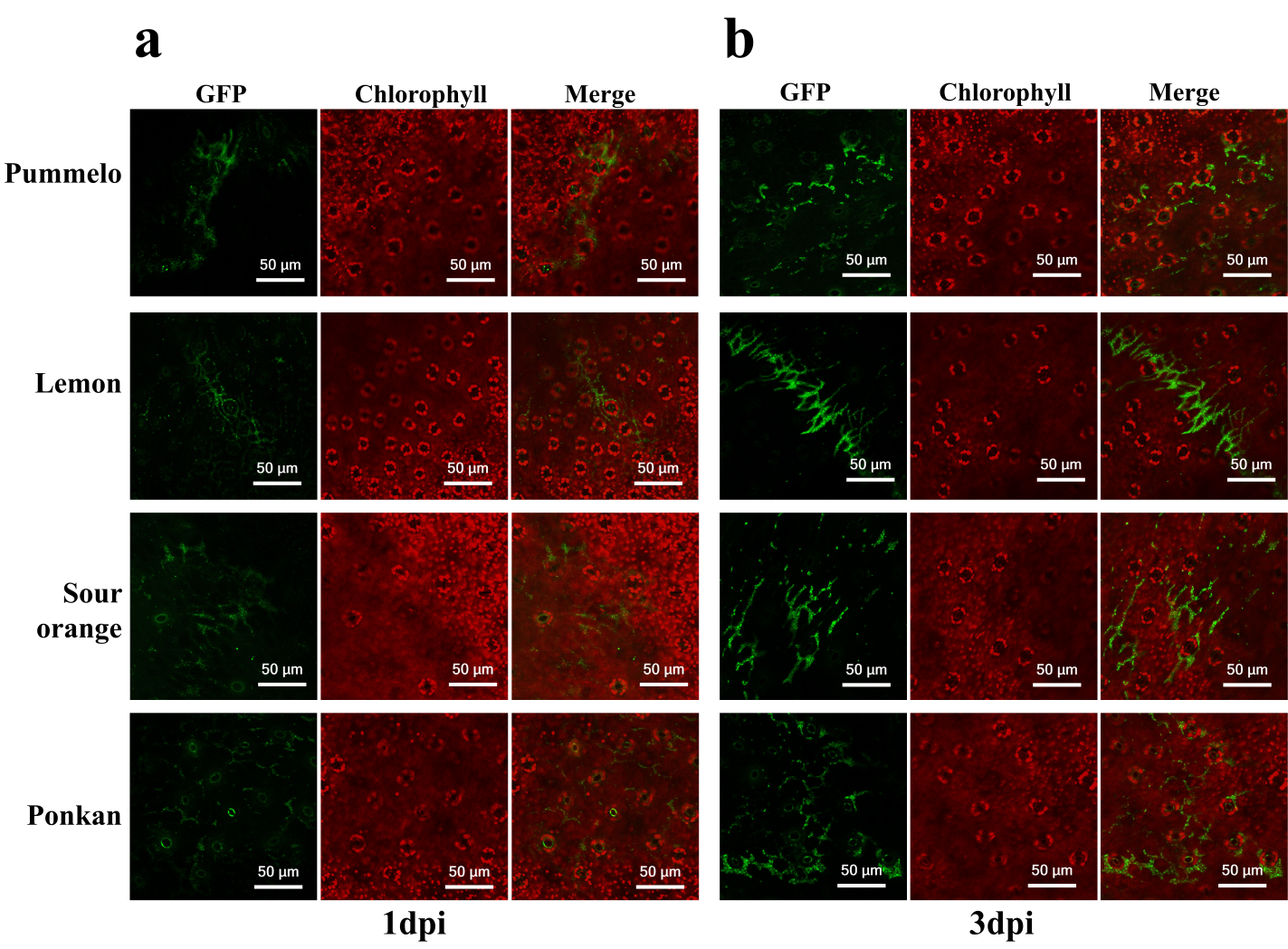


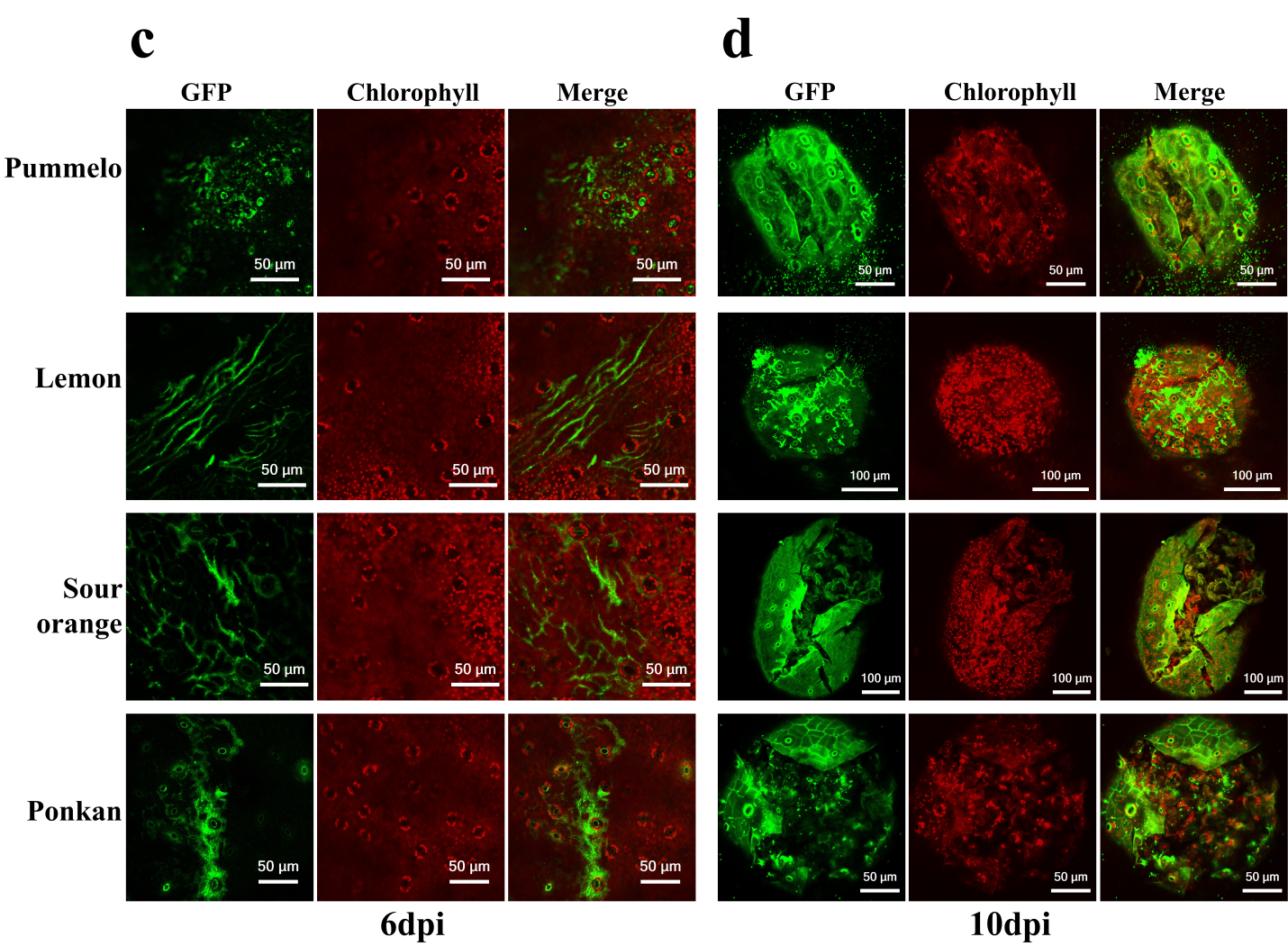


**Fig. S1** Leaf-surface growth of *eGFP*-*Xcc* in four susceptible citrus biotypes upon spray inoculation.

Fully expanded young leaves of the four indicated citrus biotypes were inoculated by spraying 10^8^ cfu/ml *eGFP*-*Xcc*. Bacterial growth on the leaf surface was monitored using CLSM. Representative images for each biotype-time point are shown. Note that strong eGFP fluorescence appeared on the inoculated leaves of all four genotypes at 10 dpi, which was concomitant with the appearance of typical canker disease symptoms (as shown in Fig. 1b).


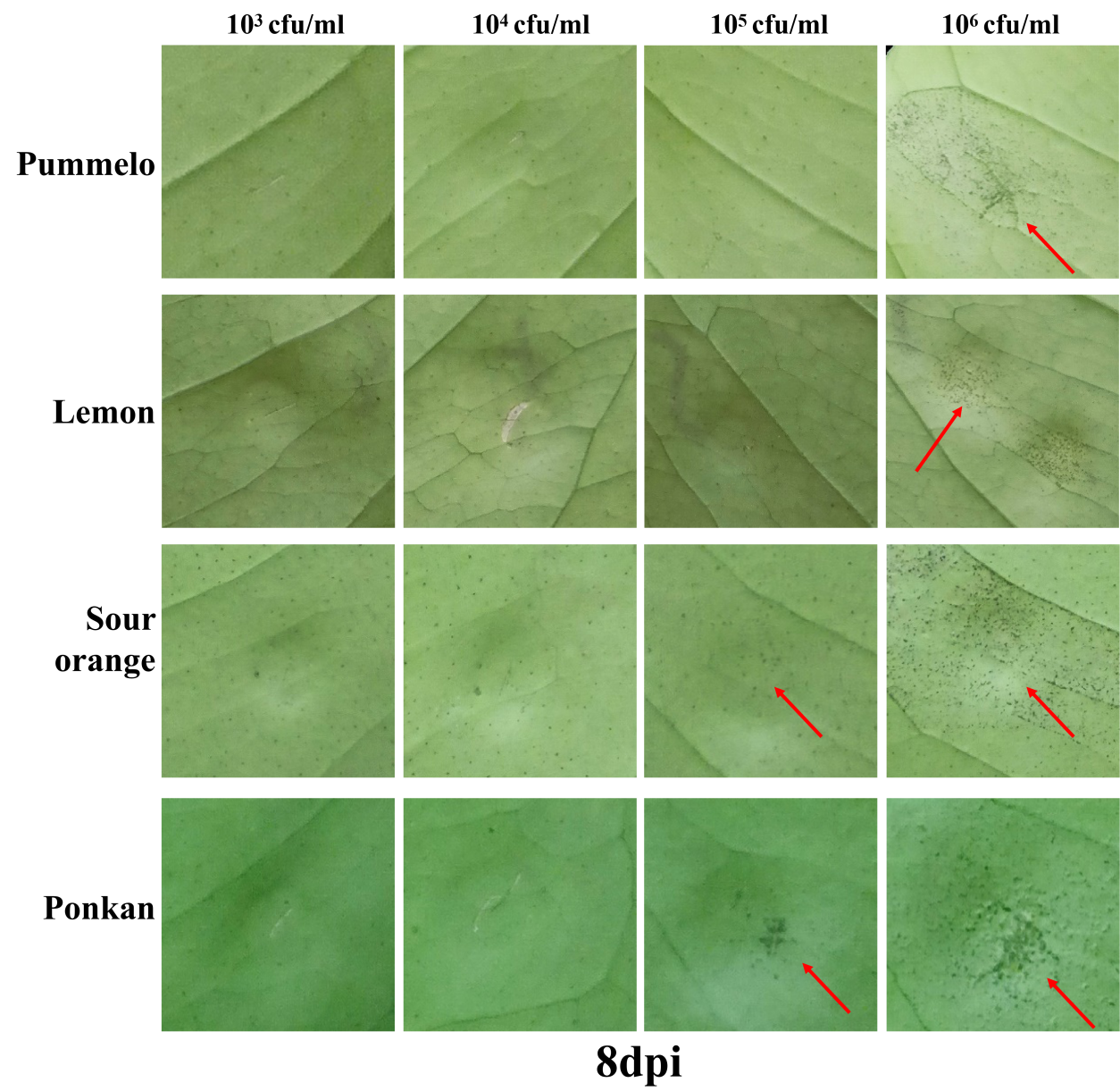


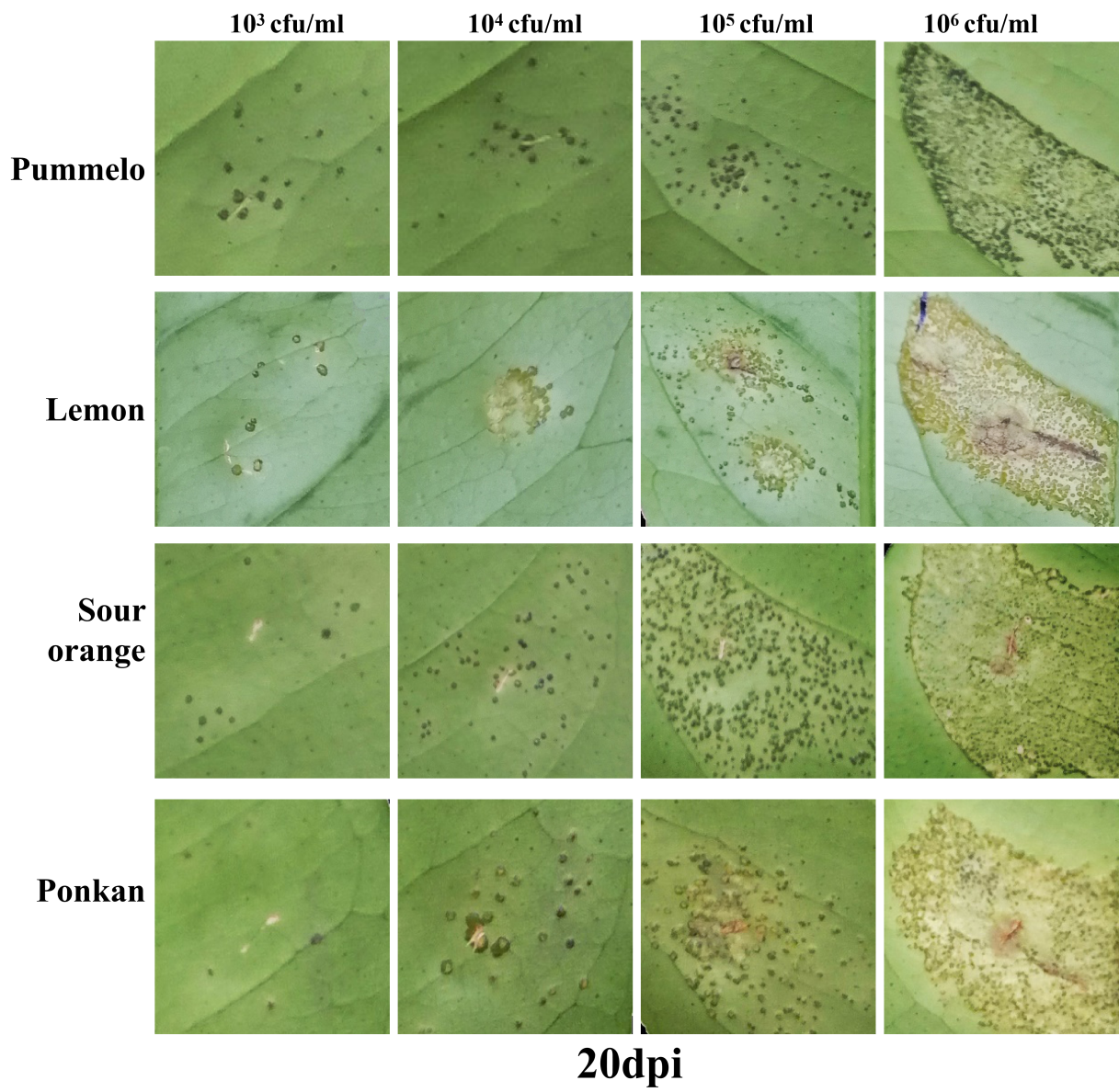


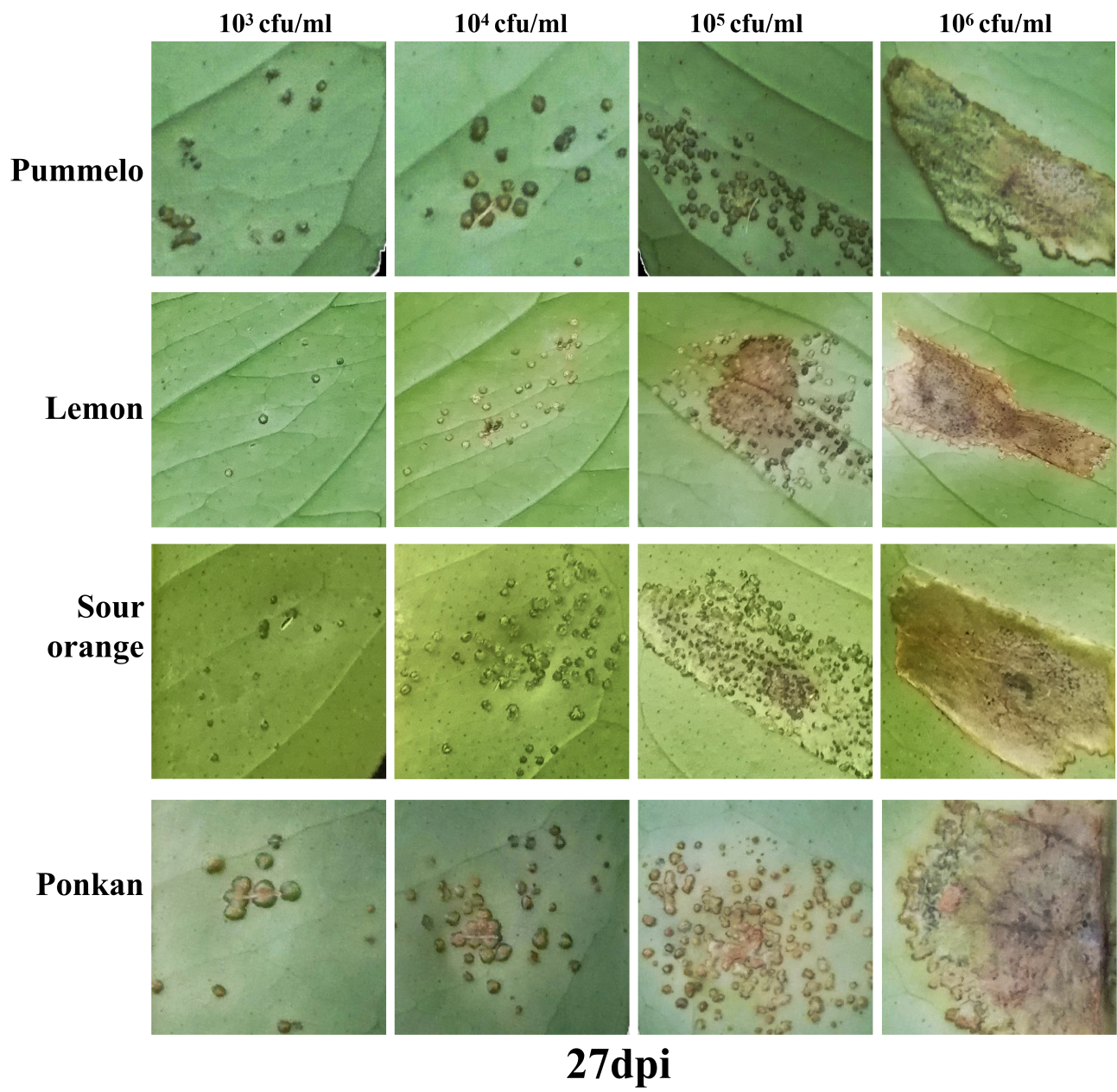


**Fig. S2** Development of canker disease symptoms on the leaves of four susceptible citrus biotypes upon infiltration with *Xcc*.

Fully expanded young leaves of the four indicated citrus biotypes were infiltrated with the four indicated bacterial concentrations. Disease development was monitored by the naked eye. Representative leaf sections at the indicated time points are shown. Note that water-soaked lesions (indicated by red arrows) were first visible at 8 dpi.


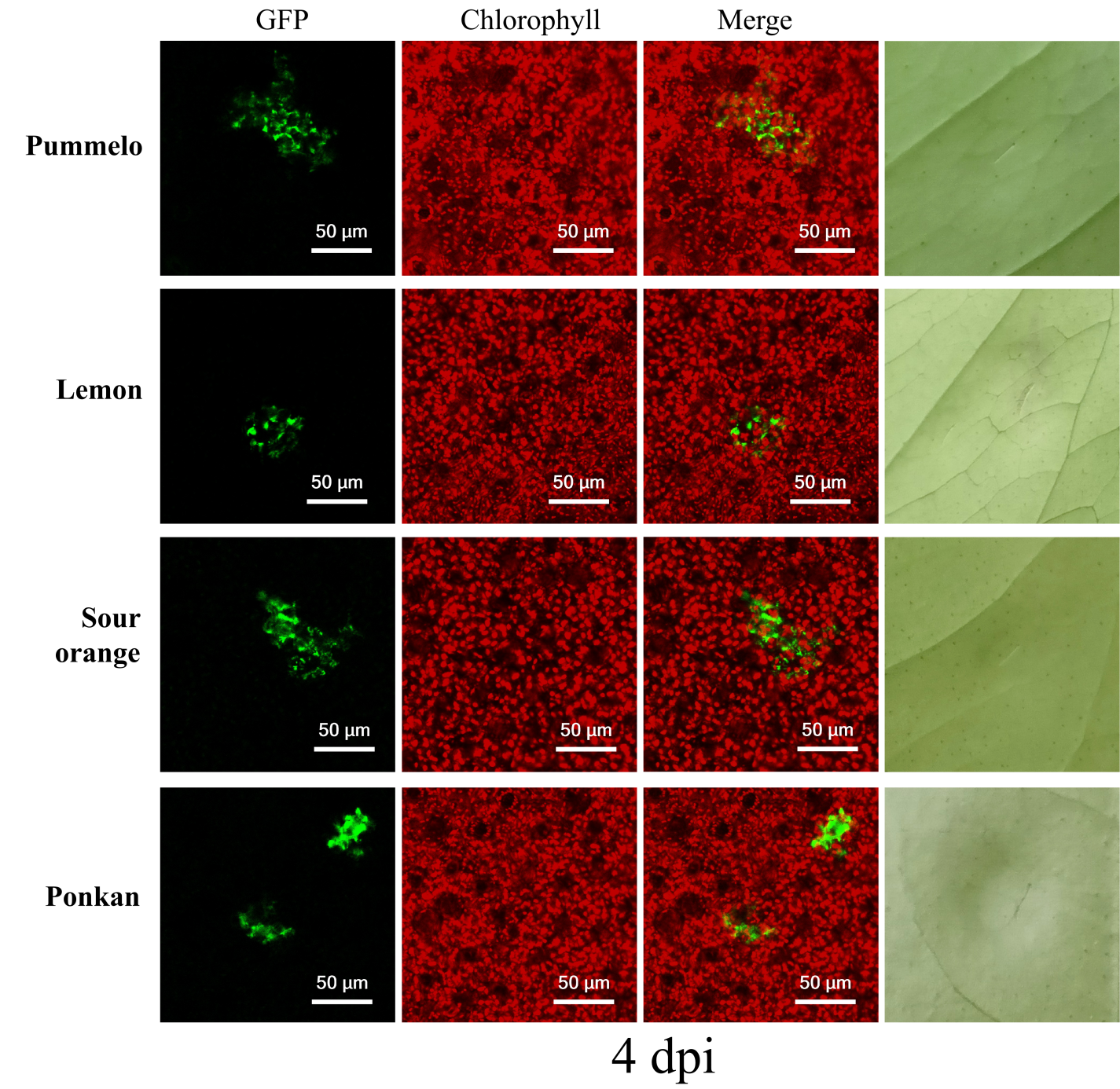


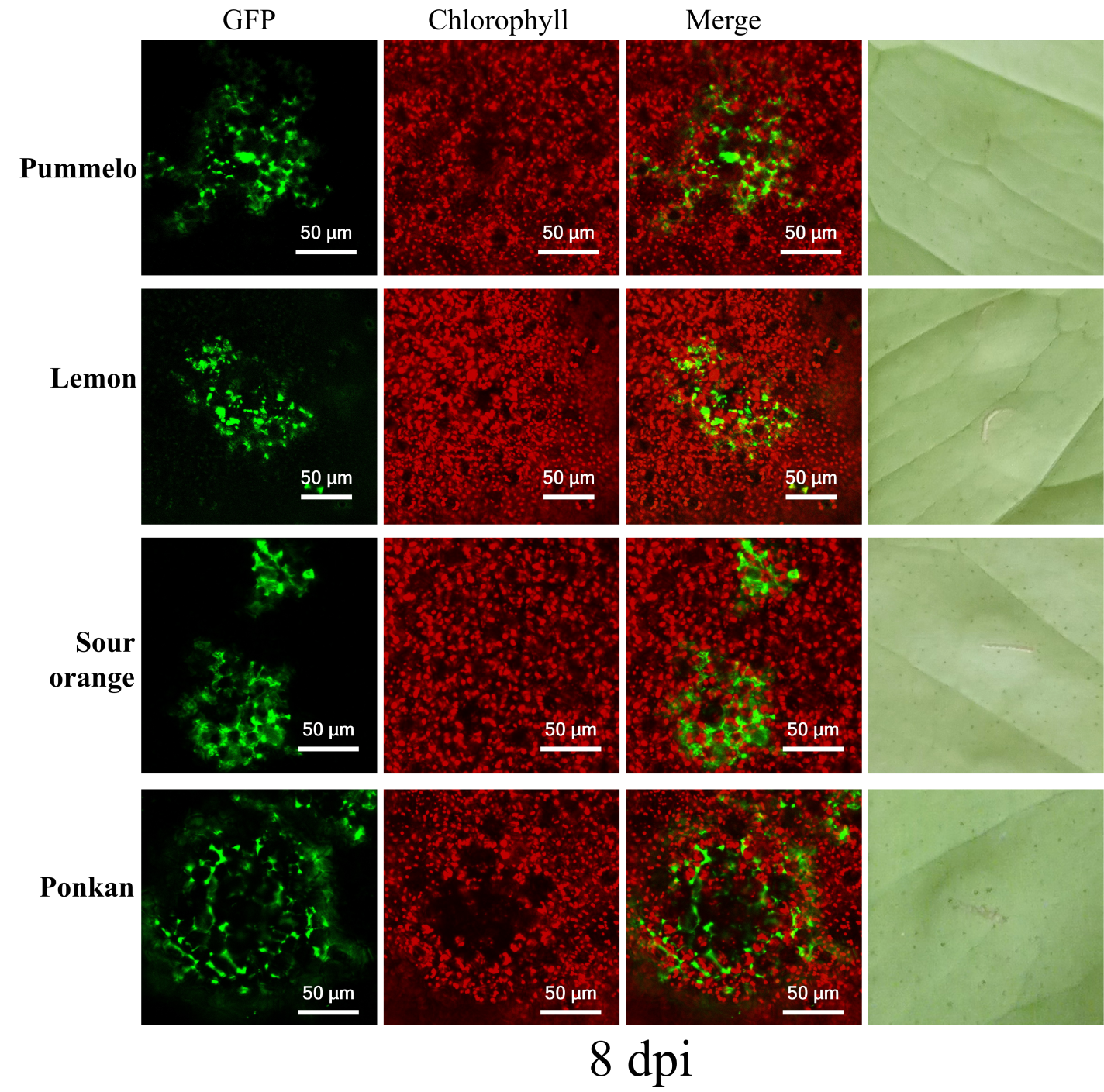


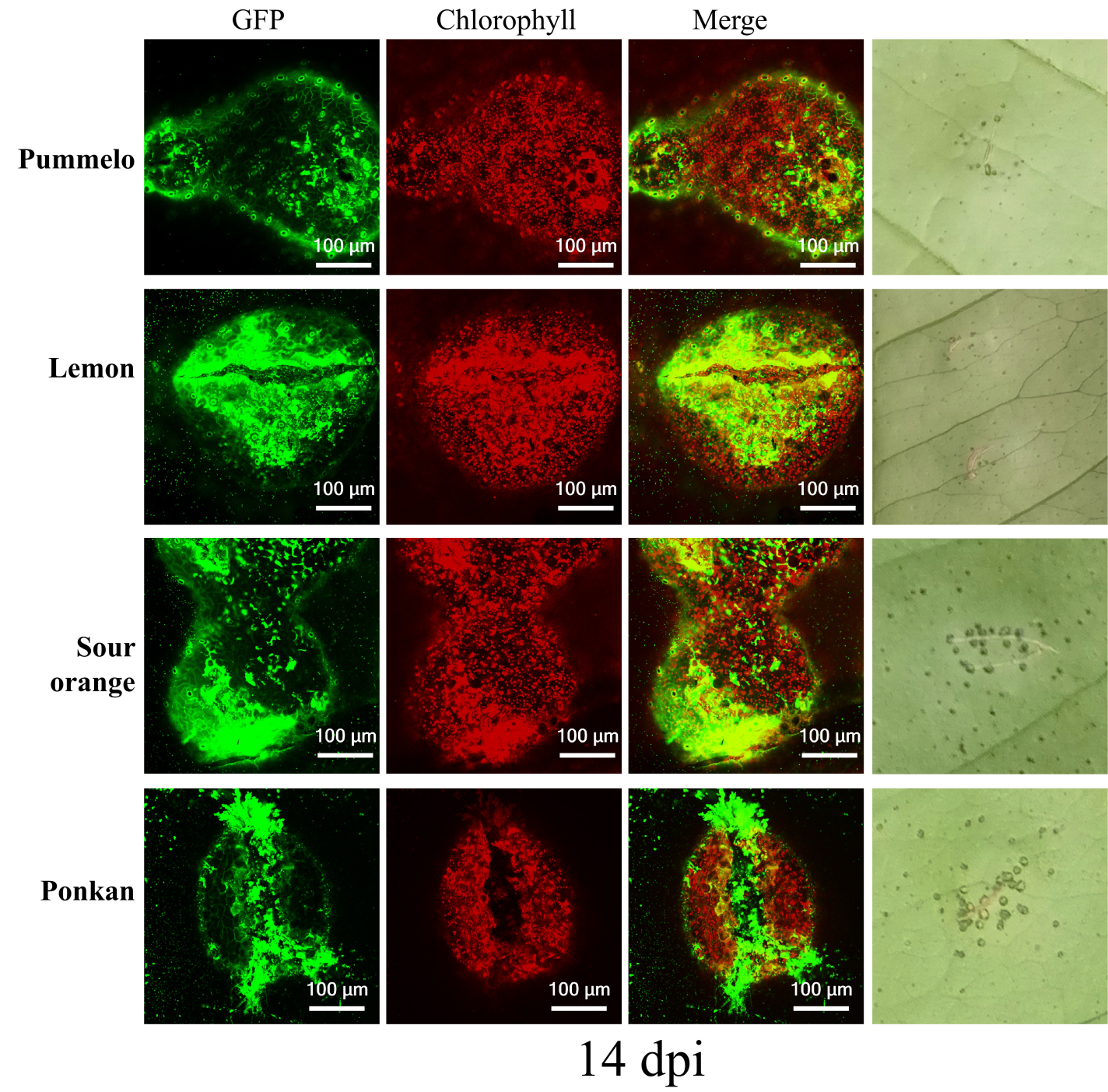


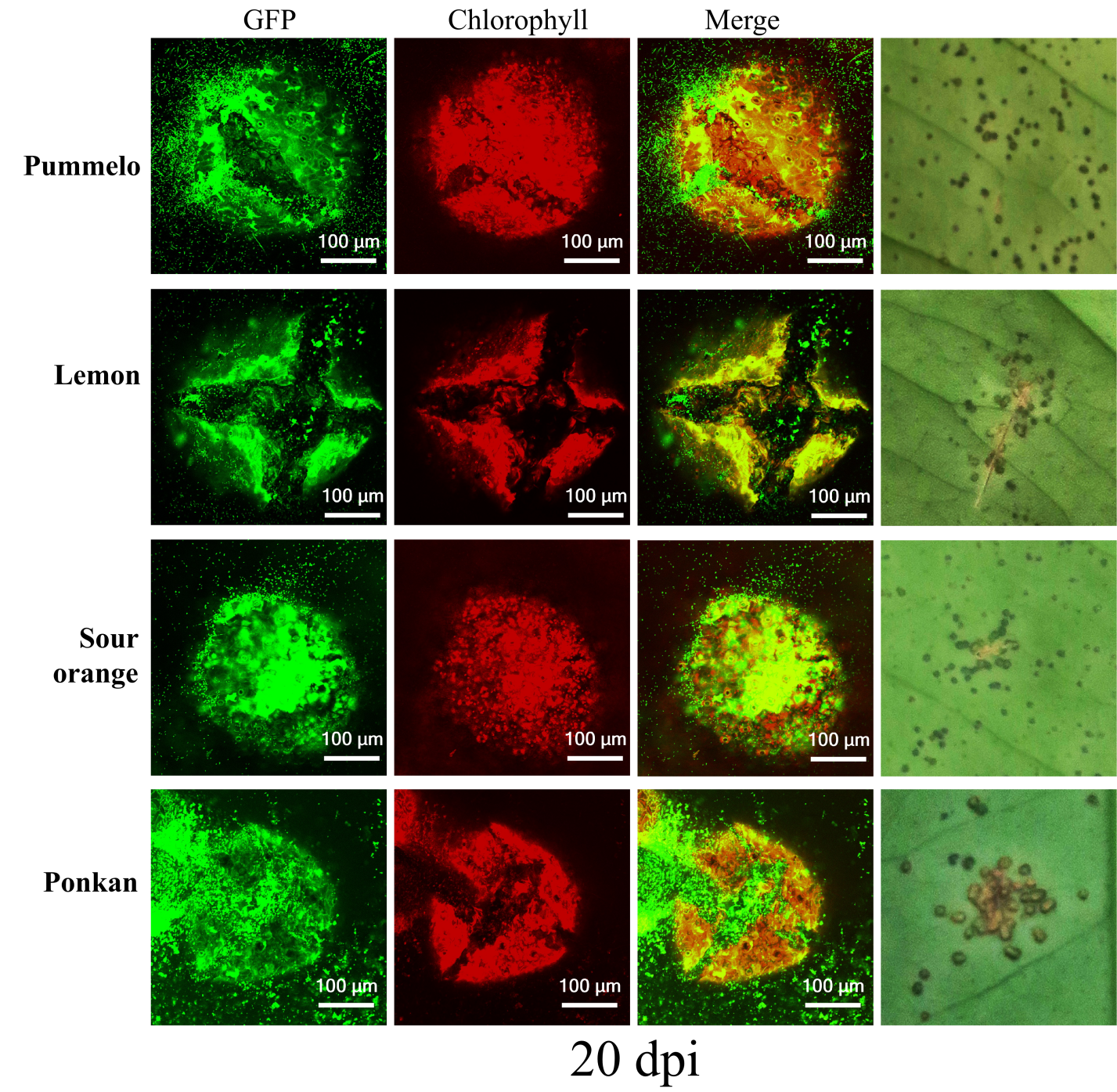


**Fig. S3** Bacterial proliferation and canker disease symptom development on the leaves of four susceptible citrus biotypes upon infiltration with *Xcc*.

Fully expanded young leaves of the four indicated citrus biotypes were infiltrated with 10^4^ cfu/ml *Xcc*. Bacterial growth in the mesophyll tissues was monitored using CLSM, and disease symptom development was monitored by the naked eye. Representative images or leaf sections at the indicated time points are shown.


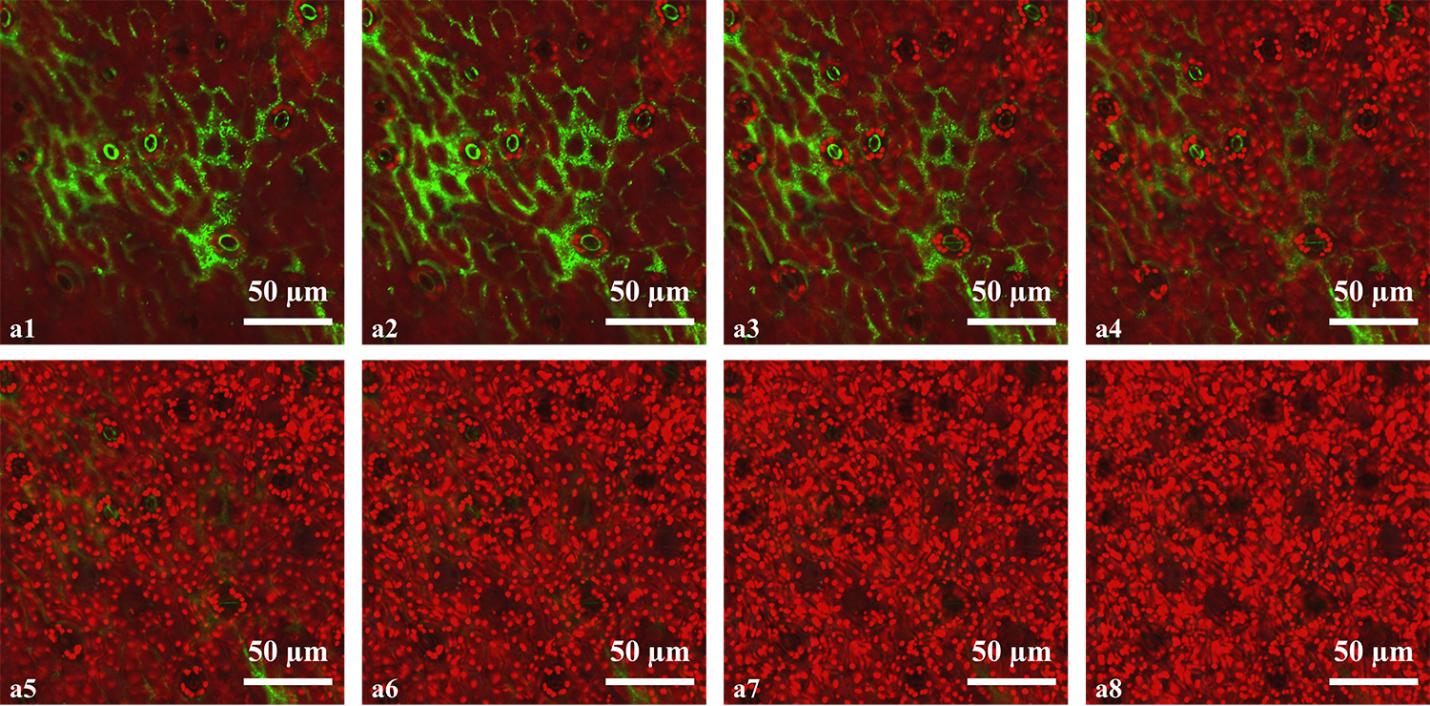


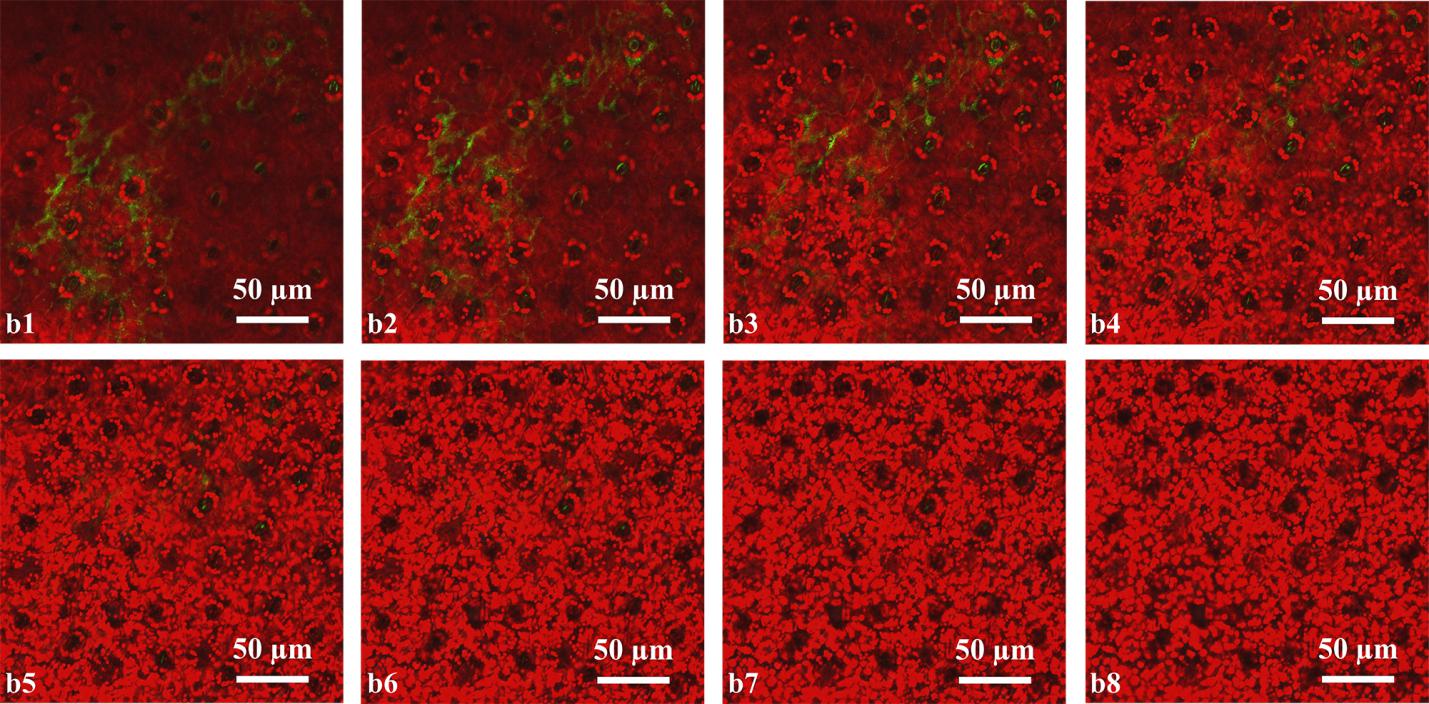


**Fig. S4** Growth of *eGFP*-*Xcc* on the leaf surface upon spray inoculation.

Leaves were inoculated by spraying 10^8^ cfu/ml *eGFP*-*Xcc*. Bacterial growth on the leaf surface was monitored using CLSM Z-stack imaging at 6 dpi. a: susceptible citrus biotypes, b: Citron C-05.
